# Prevalence of Work-Related Musculoskeletal Disorders in the Nurses Working in Hospitals of Xinjiang Uygur Autonomous Region

**DOI:** 10.1155/2017/5757108

**Published:** 2017-07-13

**Authors:** Ping Yan, Fuye Li, Li Zhang, Yi Yang, Amei Huang, Yanan Wang, Hua Yao

**Affiliations:** ^1^Graduate College, Xinjiang Medical University, Urumqi, China; ^2^Nursing Department, The First Affiliated Hospital of Xinjiang Medical University, Urumqi, China; ^3^College of Public Health, Xinjiang Medical University, Urumqi, China; ^4^Department of ICU, The First Affiliated Hospital of Xinjiang Medical University, Urumqi, China; ^5^College of Nursing, Xinjiang Medical University, Urumqi, China; ^6^Clinical Medicine Research Center, The First Affiliated Hospital of Xinjiang Medical University, Urumqi, China

## Abstract

**Objective:**

To investigate the status of work-related musculoskeletal disorders (WMSDs) in nurses working in the hospitals in Xinjiang Uygur Autonomous Region.

**Methods:**

The prevalence of WMSDs since working and in the previous 12 months was evaluated using self-administrated modified musculoskeletal questionnaire based on North European questionnaire. In this cross-sectional study, 6674 nurses involved in the nursing profession were selected from 16 hospitals using the stratified cluster sampling method.

**Results:**

The most commonly affected regions by WMSDs were lower back, neck, shoulder, and back, with an annual prevalence of 62.71%, 59.77%, 49.66%, and 39.50%, respectively. Statistical differences were noticed in the annual prevalence of WMSDs in those with different ages (*P* < 0.01) and working durations (*P* < 0.01). Logistic regression analysis indicated that the following risk factors were associated with the prevalence of WMSDs: working duration of ≥6 years; working in the Emergency Department, Department of Anesthesia, or Supply Room; night shift of more than once, working duration of >40 hrs per week; poor health status; and feeling of fatigue. Rest time of >10 min and no history of WMSDs were the protective factors of WMSDs.

**Conclusions:**

Shift and working/rest duration was closely related to WMSDs.

## 1. Introduction

Work-related musculoskeletal disorders (WMSDs), one of the major factors for the early exit from the labor market, are usually related to increasing compensation and health costs, reduced productivity, and lower quality of life [1, 2]. The major features of WMSDs included pain, discomfort, and movement limitation mainly presenting in the lower back, shoulder, neck, forearm, and hands [3]. To date, the most commonly reported symptoms were nonspecific low back pain (LBP), neck-shoulder and wrist-hand syndrome, and carpal tunnel syndrome [4].

WMSDs have been listed as occupational disorders by International Labor Organization (ILO) since 1960. In the Western countries, WMSDs rank as the second occupational disorder secondary only to the dermatergosis [5]. According to a survey, the annual prevalence of WMSDs is up to 50% in the nurses with a life-long prevalence of 35–80%, which is considered as the major cause for the decrease of working efficiency [6, 7].

In the nursing professionals, WMSDs have been considered as the leading factor for the absenteeism among the nursing professionals [8]. WMSDs have been reported to obviously affect the quality of life in nurses. According to the survey by the Bureau of Labor Statistics (BLS), WMSDs are the most common disorder in the nursing professionals. In a recent study, more than half of the nurses (54%) showed WMSDs in the lower back, followed by neck (41%), shoulder (34%), and hand-wrist (26%) [9]. Meanwhile, half of the nursing populations showed poor sleeping and working disability induced by WMSDs [10]. In studies performed in the Indian population and Portuguese population, similar results were obtained featured by a higher prevalence of WMSDs in the nursing professionals [11, 12]. In general, WMSDs hamper the working efficiency of nurses, which then affect the safety of the patients in clinical practice. Considering the importance of epidemiological knowledge related to WMSDs among the nursing professionals, it is necessary to summarize the symptoms in a broader context. To our best of knowledge, extremely rare studies have been focused on the identification of risk factors in the nurses in the WMSDs in China mainland. In this study, we aim to investigate the main symptoms presented by nurse technicians and licensed practical nurses in the Xinjiang Uygur Autonomous Region.

## 2. Materials and Methods

### 2.1. Participants

In this cross-sectional study, 6674 nurses involved in the nursing profession were selected from the hospitals in 16 Xinjiang Autonomous regions using the stratified cluster sampling method. The inclusion criteria were as follows: the licensed practical nurses with a working experience of more than 12 months in the permanent position. The exclusion criteria were as follows: (i) those with MSD caused by congenital spine disorders, cancer, trauma, and gynecological disease; (ii) those with pain due to surgery, tumor vessel lesions, irregular menstruation cycle, scoliosis, disc protrusion, spine malformation, and ankylosing spondylitis; (iii) those with a long-term administration of analgesics; and (iv) those with a history of psychiatric disorder. Informed consent was obtained from each subject. The study protocols were approved by the Ethical Committee of the First Affiliated Hospital of Xinjiang Medical University.

All the participants were required to fill in the modified self-administrated musculoskeletal questionnaire in person [13].

### 2.2. Measures

The following information was covered by the questionnaire: (i) demographics, such as working/resting duration, shift (day or night), and frequency of night shift; (ii) work-related factors, including gender, age, race, body mass index, academic degree, and working department; and (iii) health related factors such as physical activity and/or history of sick leave. The following regions of the body were included in the survey in the questionnaire: back, shoulder, neck, elbow, lower back, hand/wrist, hip, knee, and ankle/foot. WMSD was diagnosed based on the previous description [14]. The Chinese version of musculoskeletal questionnaire was used to identify the symptoms in any part in the previous 12 months [13].

### 2.3. Statistical Analysis

The entry of the information collected from the questionnaire was entered into the EpiData. SPSS 21.0 software was used for the data analysis. The qualitative data were descriptively analyzed by calculating the percentage or frequencies, and quantitative data were presented as mean ± standard deviation (SD). Logistic regression analysis was used to identify the risk factors for WMSDs and the assignment of the risk factors was listed in Table 1. *P* < 0.05 was considered to be statistically significant.

## 3. Results

### 3.1. Subject Characteristics

In total, 6674 active nurses (6674/6899, 95.51%; male: 214; female: 6460; aged: 16–54 yrs, mean age: 31.83 ± 7.18) accomplished the questionnaire. The working time in the institution ranged from 1 to 35 yrs (mean: 10.35 ± 7.84 yrs). The BMI was 14.52–53.99 kg/m^2^ (21.81 ± 3.03 kg/m^2^). Among these participants, 4426 (66.32%) were Han Chinese, while the others were minorities. For the academic degree, 1933 (28.96%) were with at least bachelor degree. For the health conditions, the numbers of participants with satisfactory, moderate, poor, or extremely poor physical activities were 1032 (15.46%), 4223 (63.28%), 1177 (17.46%), and 242 (3.63%), respectively. The working time per week was 30–65 hrs (mean: 46.87 ± 4.42 hrs) per week.

### 3.2. Prevalence of WMSDs and Sick Leave

The prevalence of WMSDs since working and in the previous 12 months was 81.18% and 77.43%, and the prevalence of sick leave was 10.50% and 9.39%, respectively. The prevalence of two or more body regions with WMSDs was 72.83% and 68.03%. The most commonly affected regions were lower back, neck, shoulder, and back, with an annual prevalence of 62.71%, 59.77%, 49.66%, and 39.50%, respectively. The sick leave due to WMSDs ever since the occupation was mainly associated with symptoms in back (6.17%), neck (3.06%), ankle (2.10%), and knee (1.68%). The prevalence of sick leave within the 12 months was 5.50% (wrist), 2.38% (neck), 1.56% (ankle), and 1.18% (shoulder), respectively (Table 2).

### 3.3. Annual Prevalence of WMSDs in Nursing Professionals with Different Ages

Among the nurses with different ages, statistical differences were noticed in the prevalence of WMSDs in the recent 12 months and in 4 regions of the body including lower back, neck, shoulder, and back (*P* < 0.05). Chi-squared statistics revealed that age was significantly associated with the prevalence of WMSDs in the recent 12 months (*χ*^2^ = 289.885, *P* < 0.01) and the prevalence of WMSDs in lower back (*χ*^2^ = 168.715, *P* < 0.01), neck (*χ*^2^ = 250.122, *P* < 0.01), shoulder (*χ*^2^ = 152.239, *P* < 0.01), and back (*χ*^2^ = 108.742, *P* < 0.01). Compared with the subjects aged less than 25 yrs, remarkable increase was noticed in the prevalence of WMSDs in those aged 26–30 yrs (*χ*^2^ = 215.843, *P* < 0.01), 31–35 yrs (*χ*^2^ = 243.427, *P* < 0.01), 36–40 yrs (*χ*^2^ = 205.850, *P* < 0.01), and ≥41 yrs (*χ*^2^ = 266.530, *P* < 0.01). Compared with the subjects aged 26–30 yrs, remarkable increase was noticed in the prevalence of WMSDs in those aged 31–35 yrs (*χ*^2^ = 9.640, *P* < 0.01) and 36–40 yrs (*χ*^2^ = 34.502, *P* < 0.01) and those aged ≥41 yrs (*χ*^2^ = 41.915, *P* < 0.01). Meanwhile, compared with those aged 31–35 yrs, remarkable increase was identified in the prevalence of WMSDs in those aged 36–40 yrs (*χ*^2^ = 14.652, *P* < 0.01), and those aged ≥41 yrs (*χ*^2^ = 15.172, *P* < 0.01). Among the subjects aged 36–40 yrs, the most commonly affected regions were lower back and back, while, in those aged ≥41 yrs, the most affected regions were neck and shoulder (Table 3).

### 3.4. Annual Prevalence of WMSDs in Nursing Professionals with Different Working Time in the Departments

Statistical differences were noticed in the annual prevalence of WMSDs in the nurses with various working time in the institution in the recent 12 months, and the regions in the lower back, neck, shoulder, and back (*P* < 0.05). Chi square test revealed the annual prevalence of WMSDs increased with the age in the recent 12 months (*χ*^2^ = 254.504, *P* < 0.01), and the regions in the lower back (*χ*^2^ = 165.218, *P* < 0.01), neck (*χ*^2^ = 224.450, *P* < 0.01), shoulder (*χ*^2^ = 143.822, *P* < 0.01), and back (*χ*^2^ = 107.393, *P* < 0.01). Compared with those with a working time of less than 5 yrs, remarkable increase was observed in the annual prevalence of WMSDs in those with a working time of 6–10 yrs (*χ*^2^ = 159.381, *P* < 0.01), 11–15 yrs (*χ*^2^ = 134.476, *P* < 0.01), 16–20 yrs (*χ*^2^ = 113.865, *P* < 0.01), and 21 yrs or more (*χ*^2^ = 169.587, *P* < 0.01). Compared with those with a working time of 6–10 yrs, remarkable increase was observed in the annual prevalence of WMSDs in those with a working time of 16–20 yrs (*χ*^2^ = 19.448, *P* < 0.01) and 21 yrs or more (*χ*^2^ = 21.585, *P* < 0.01). Meanwhile, statistical difference was observed in the annual prevalence of WMSDs in those with a working time of 11–15 yrs compared with those with a working time of 16–20 yrs (*χ*^2^ = 9.269, *P* < 0.01). For the subjects with a working time of 16–20 years, the highest prevalence of WMSDs was noticed in the recent 12 months, lower back and back, while, in those with a working time of 21 or more, the most affected body regions were neck and shoulder (Table 4).

### 3.5. Annual Prevalence of WMSDs in Different Departments

Statistical differences were noticed in the annual prevalence of WMSDs in the nurses in different departments in the recent 12 months, and the regions in the lower back, neck, shoulder, and back (*P* < 0.05). Among the nurses working in the Department of Internal Medicine, Department of Surgery, Department of Emergency, and Department of Anesthesia, statistical differences were noted in those working in the prevalence of WMSDs. The prevalence of WMSDs was the highest in the Department of Emergency, while the prevalence was the lowest in the Internal Medicine Department (Table 5).

### 3.6. Annual Prevalence of WMSDs in Nurses with Different Working Times

The annual prevalence of WMSDs increased in the lower back, neck, shoulder, and back of the nurses with the increase of the working duration (*P* < 0.01). Compared with those with a working time of 40 hr per week, significant increase was identified in the prevalence of WMSDs in those with a time of 41–50 hrs (*χ*^2^ = 40.187, *P* < 0.01) and with a time of ≥51 hrs (*χ*^2^ = 17.135, *P* < 0.01, Table 6).

### 3.7. Identification of Risk Factors for the WMSDs

Logistic regression analysis was performed using the variables including age, working time in the institution, gender, race, BMI, academic degree, health conditions, shift, and working times per week. The results indicated a working time of ≥6 yrs, working in the Emergency Department, Department of Anesthesia, Department of Supply Room, shift, night shift of more than 1 per week, working time of ≥40 hrs per week, poor health conditions, and fatigue were the risk factors of WMSDs. In contrast, rest during the shift, a rest lasting for >10 min and no disease history of WMSDs were the protective factors for WMSDs (Table 7).

## 4. Discussion

 Individuals involved in the nursing profession are more susceptible to the WMSDs. According to a previous study, excessive work load and nonstandard work posture were the risk factors for the WMSDs [15]. In a national survey, the annual prevalence of WMSDs and the weekly prevalence of WMSDs were 93% and 64.1%, respectively [16]. In China, the reported prevalence of WMSDs was in a range of 56.62% to 78.6% [17]. In this study, the prevalence of WMSDs and the weekly prevalence of WMSDs were 77.43% and 44.79%, respectively. Besides, the prevalence was higher in the lower back (62.71%), neck (59.77%), shoulder (49.66%), and back (39.50%). In Sudary region, the frequency of low back bending (such as long pushing or pulling) and activity limitation was high in the nursing professionals [18]. Particularly, the carrying and lifting factors, such as changing the bed sheet and body turn-over of patients, may induce the incidence of OMSI. Besides, a large number of nurses may suffer from injury during patient transfer. In the past decades, principles for patient transfer were issued by the Royal Nursing School and American Nurse Association (ANA) in order to prevent the potential injuries to the nurses during the patient transfer, as well as reducing the incidence of low back pain and the development and/or recurrence of WMSDs [19]. In China, no such guidelines have been established by the government or local authorities.

In this study, the ages of the nurses were identified as the risk factor of WMSDs. Aged nurses showed a longer working duration (*r* = 0.917, *P* < 0.01). Despite the fact that age was not entered into the regression equation, it was considered that the incidence of WMSDs may increase with the increase of the age and working duration [20]. Besides, the work load may increase with the extension of the work duration, and the work load increased in the presence of compulsive position, which may finally induce the increase of prevalence of WMSDs [11]. Moreover, the interruption of the work load and working balance of the body may contribute to the generation of chronic overload, which may be also a potential factor for the pathogenesis of WMSDs.

Nurses working in the Emergency Department showed the highest prevalence of WMSDs, followed by those working in the Supply Room, Department of Surgery, and Department of Anesthesia. In the Emergency Department, the nurses were involved in frequent lifting and pushing in the clinical practice. Besides, these individuals were exposed to a long-term high stress, which may induce WMSDs. For the individuals in the Supply Room, they were frequently involved in long-term standing, persistent head bowing, and frequent transfer of the medical facilities. For the nurses in the Operating Room, the work load in the local muscles increased under a static condition, which may induce interruption in the blood circulation. The fatigue of muscles and bones caused by frequent lifting and pushing was an important risk factor of low back pain in the nurses. Simultaneously, long-term high pressure and fast working frequency may induce sever muscle injuries.

Working time of 50 hrs per week and a daily working time of 8.5 hrs were considered as a risk factor for the WMSDs. In this study, the working time of the nurses in our hospital was in a range of 30–65 hrs and the weekly time was 46.87 ± 4.42. According to the multivariate analysis, adequate rest was a protective factor for the WMSDs. In a previous study, Wang et al. revealed that a rest time of 15 min could guarantee the recovery of erector muscle of spine and heart rate into the normal range [21]. Therefore, adequate rest is recommended for the nurses involved in the nursing profession [6], in order to attenuate the stress and relax the muscle tissues, as well as eliminate the fatigue of muscles.

There are limitations in our study. Despite the fact that a large number of nurses working in the Xinjiang Autonomous Region were included in this study, the sample number is not large enough to bring completely accurate data for the WMSDs in the nurses. Meanwhile, it is not adequate to establish a system to prevent the incidence of WMSDs in the nurses. In future, we will focus on the study of how to prevent the WMSDs in the nursing population.

## 5. Conclusions

In this study, we aim to investigate the status of WMSDs in the nurses in the Xinjiang Autonomous Region. Our data indicated that shift and working/rest duration were closely related to the prevalence of WMSDs. In future, further measures should be taken to arrange for appropriate shift and rest in order to decrease the incidence of WMSDs.

## Figures and Tables

**Table 1:** Assignment of risk factors for WMSD of the nurses.

Factor	Score
Sex	0 = M; 1 = F
Age (yr)	*0 = ≤25 yrs*; 1 = 26–30 yrs; 2 = 31–35 yrs; 3 = 36–40 yrs; 4 = ≥41 yrs
Working duration (yr)	*0 = ≤5 yrs*; 1 = 6–10 yrs; 2 = 11–15 yrs; 3 = 16–20 yrs; 4 = ≥21 yrs
BMI (kg/m^2^)	*0 = *<*18.5*; 1 = 18.5~23.9; 2 = 24.0~27.9; *3 = ≥28.0*
Department	
Department of Internal Medicine	$\begin{array}{ccccc} 0 & 0 & 0 & 0 & 0 \end{array}$
Department of Surgery	$\begin{array}{ccccc} 0 & 1 & 0 & 0 & 0 \end{array}$
Department of Emergency	$\begin{array}{ccccc} 0 & 0 & 1 & 0 & 0 \end{array}$
Department of Anesthesia	$\begin{array}{ccccc} 0 & 0 & 0 & 1 & 0 \end{array}$
Supply room	$\begin{array}{ccccc} 0 & 0 & 0 & 0 & 1 \end{array}$
Race	1 = Han Chinese; 2 = Minorities
Frequency of rest during working	Based on the exact working duration in the nursing care
Accumulated rest time	*0* < 10 min; 1 = 10~30 min; 2 = >30 min
Shift	0 = No; 1 = Yes
Night shift	*0 = None*; 1 = 1–5; 2 = 6–9; 3 = ≥10
Previous history	0 = None; 1 = Yes
Working time per week	Based on the exact working time in the nursing care
Healthy conditions	*0 = Satisfactory*; 1 = general; 2 = poor; 3 = very poor
Feeling tired	*0 = Not at all*; 1 = a little; 2 = tired; 3 = very tired

The italic font served as control.

**Table 2:** Prevalence of WMSDs and sick leave of the nurses in hospitals in Xinjiang Autonomous Region.

Position	Working occupation-now		In the recent 1 year	
	Number of patients (%)	Number of patients asking for sick leave (%)	Number of patients (%)	Number of patients asking for sick leave (%)
Neck	4274 (64.04)	204 (3.06)	3989 (59.77)	159 (2.38)
Shoulder	3627 (54.35)	106 (1.59)	3314 (49.66)	79 (1.18)
Back	2940 (44.05)	77 (1.15)	2636 (39.50)	78 (1.17)
Elbow	1035 (15.51)	36 (0.54)	967 (14.49)	32 (0.48)
Lower back	4509 (67.56)	412 (6.17)	4185 (62.71)	367 (5.50)
Wrist	1650 (24.72)	60 (0.90)	1448 (21.70)	52 (0.78)
Hip	1517 (22.73)	68 (1.20)	1362 (20.41)	62 (0.93)
Knee	2477 (37.11)	112 (1.68)	2226 (33.35)	87 (1.30)
Ankle	2267 (33.97)	140 (2.10)	1993 (29.86)	104 (1.56)
WMSDs involving one body part	5418 (81.18)	701 (10.50)	5168 (77.43)	627 (9.39)
WMSDs involving at least two body parts	4861 (72.83)	242 (3.63)	4540 (68.03)	198 (2.97)

**Table 3:** Annual prevalence of WMSDs in the nurses.

Age	*N* (%)	In the recent 1 year	Lower back	Neck	Shoulder	Back
		*N* (%)	*N* (%)	*N* (%)	*N* (%)	*N* (%)
≤25	944 (14.14)	497 (52.65)	383 (40.57)	359 (38.03)	299 (31.67)	224 (23.73)
26–30	2693 (40.35)	2095 (77.79)^*∗*^	1690 (62.76)	1563 (58.04)	1311 (48.68)	1054 (39.14)
31–35	1586 (23.76)	1297 (81.7)^*∗*,†^	1071 (67.53)	1013 (63.87)	837 (52.77)	657 (41.42)
36–40	567 (8.50)	503 (88.71)^*∗*,†,∆^	412 (72.66)	401 (70.72)	329 (58.02)	282 (49.74)
≥41	884 (13.25)	776 (87.78)^*∗*,†,∆^	629 (71.15)	653 (73.87)	538 (60.86)	419 (47.40)

Total	6674 (100.00)	5168 (77.43)	4185 (62.71)	3989 (59.77)	3314 (49.66)	2636 (39.50)

*χ* ^2^	—	444.682	264.552	301.369	189.571	148.797
*P*	—	<0.001	<0.001	<0.001	<0.001	<0.001

^*∗*^ *P* < 0.01, compared with the nurses aged ≤25 yrs; ^†^*P* < 0.01, compared with the nurses aged 26–30 yrs; ^∆^*P* < 0.01, compared with the nurses aged 31–35 yrs.

**Table 4:** Annual prevalence of WMSDs in the nurses.

Working duration	*N* (%)	In the recent 1 year	Lower back	Neck	Shoulder	Back
		*N* (%)	*N* (%)	*N* (%)	*N* (%)	*N* (%)
≤5	2020 (30.27)	1283 (63.51)	995 (49.26)	946 (46.83)	781 (38.66)	610 (30.20)
6–10	2260 (33.86)	1825 (80.75)^*∗*^	1486 (65.75)	1375 (60.84)	1159 (51.28)	924 (40.88)
11–15	1107 (16.59)	922 (83.29)^*∗*,†^	778 (70.28)	725 (65.49)	595 (53.75)	468 (42.28)
16–20	447 (6.70)	400 (89.49)^*∗*,†,∆^	325 (72.71)	324 (72.48)	265 (59.28)	228 (51.01)
≥21	840 (12.59)	738 (87.86)^*∗*,†,∆^	601 (71.55)	619 (73.69)	514 (61.19)	406 (48.33)

Total	6674 (100.00)	5168 (77.43)	4185 (62.71)	3989 (59.77)	3314 (49.66)	2636 (39.55)

*χ* ^2^	—	349.312	239.548	254.520	168.734	130.721
*P*	—	<0.001	<0.001	<0.001	<0.001	<0.001

^*∗*^ *P* < 0.01, compared with the nurses with a working duration of ≤5 yrs; ^†^*P* < 0.01, compared with the nurses with a working duration of 6–10 yrs; ^∆^*P* < 0.01, compared with the nurses with a working duration of 11–15 yrs.

**Table 5:** Annual prevalence of WMSDs in the nurses working in different department.

Department	*N* (%)	In the recent 1 year	Lower back	Neck	Shoulder	Back
		*N* (%)	*N* (%)	*N* (%)	*N* (%)	*N* (%)
Department of Internal Medicine	2297 (34.42)	1733 (75.45)	1352 (58.86)	1325 (57.68)	1076 (46.84)	825 (35.92)
Department of Surgery	2223 (33.31)	1732 (77.91)	1410 (63.43)	1335 (60.05)	1123 (50.52)	888 (39.95)
Department of Emergency	1182 (17.71)	945 (79.95)^*∗*^	821 (69.46)	724 (61.25)	622 (52.62)	541 (45.77)
Department of Anesthesia	443 (6.64)	340 (76.75)	264 (59.59)	260 (58.69)	208 (46.95)	160 (36.12)
Supply room	529 (7.93)	418 (79.02)	338 (63.89)	345 (65.22)	285 (53.88)	222 (41.97)

Total	6674 (100.00)	5168 (77.43)	4185 (62.71)	3989 (59.77)	3314 (49.66)	2636 (39.50)

*χ* ^2^	—	10.643	40.230	12.054	17.150	35.441
*P*	—	0.031	<0.001	0.017	0.002	<0.001

^*∗*^ *P* < 0.01 compared with the nurses working in the Department of Internal Medicine.

**Table 6:** Prevalence of WMSDs in the nurses with various working duration per week.

Working duration per week	*N* (%)	In the recent 1 year	Lower back	Neck	Shoulder	Back
		*N* (%)	*N* (%)	*N* (%)	*N* (%)	*N* (%)
≤40	974 (14.59)	676 (69.40)	516 (52.98)	497 (51.03)	434 (44.56)	354 (36.34)
41~50	5366 (80.40)	4221 (78.66)^*∗*^	3432 (63.96)	3261 (60.77)	2671 (49.78)	2110 (39.32)
≥51	334 (5.00)	271 (81.14)^*∗*^	237 (70.96)	231 (69.16)	209 (62.5)	172 (51.50)

Total	6674 (100.00)	5168 (77.43)	4185 (62.71)	3989 (59.77)	3314 (49.66)	2636 (39.50)

*χ* ^2^	—	43.191	52.743	45.455	32.454	24.245
*P*	—	<0.001	<0.001	<0.001	<0.001	<0.001

^*∗*^ *P* < 0.05, compared with nurses with a working duration of less than 40 hrs per week.

**Table 7:** Logistic regression analysis of the risk factors of WMSDs in nurses.

Factor	*P*	OR (95% CI)
Working duration	<0.001	
6–10	<0.001	2.131 (1.834–2.475)
11–15	<0.001	2.829 (2.324–3.445)
16–20	<0.001	5.368 (3.823–7.537)
≥21	<0.001	6.899 (5.281–9.011)
Department	0.001	
Department of Surgery	0.056	1.156 (0.997–1.340)
Department of Emergency	0.003	1.331 (1.104–1.605)
Department of Anesthesia	0.002	1.524 (1.164–1.995)
Supply room	0.008	1.436 (1.100–1.874)
Frequency of rest during working	<0.001	0.792 (0.719–0.871)
Accumulated working time	<0.001	
<10 min	<0.001	0.338 (0.251–0.455)
10–30 min	<0.001	0.429 (0.323–0.569)
>30 min	<0.001	0.115 (0.072–0.183)
Shift	<0.001	2.479 (1.877–3.274)
Night shift per month	<0.001	
1–5 times	<0.001	2.606 (2.082–3.262)
6–9 times	<0.001	3.654 (2.941–4.539)
≥10 times	<0.001	3.763 (2.996–4.728)
With no previous disease history	<0.001	0.646 (0.563–0.742)
Working time per week	<0.001	1.376 (1.186–1.597)
Health status	<0.001	1.587 (1.429–1.764)
Work-related fatigue	<0.001	1.431 (1.314–1.559)

## Abbreviations

WMSDs: Work-related musculoskeletal disorders
NMQ: Nordic Musculoskeletal Questionnaire.
